# High Avidity Cytotoxic T Lymphocytes Can Be Selected into the Memory Pool but They Are Exquisitely Sensitive to Functional Impairment

**DOI:** 10.1371/journal.pone.0041112

**Published:** 2012-07-19

**Authors:** Victoria A. Brentville, Rachael L. Metheringham, Barbara Gunn, Lindy G. Durrant

**Affiliations:** 1 Scancell Holdings plc, Academic Department of Clinical Oncology, City Hospital Campus, University of Nottingham, Nottingham, United Kingdom; 2 Academic Department of Clinical Oncology, City Hospital Campus, University of Nottingham, Nottingham, United Kingdom; MRC National Institute for Medical Research, United Kingdom

## Abstract

High avidity cytotoxic T lymphocytes **(**CTL) are important in viral clearance and anti-tumor immunity, however, mechanisms for their optimal generation and maintenance *in vivo* remain unclear. Immunizing mice with an antibody-DNA vaccine encoding a single CTL epitope, induces a 100 fold higher avidity response than peptide vaccination with the identical epitope. The high avidity response is retained into memory and can be efficiently reactivated with an antibody-DNA boost. In contrast, reactivation of high avidity CTL with peptide, stimulated responses with a significant drop in avidity, suggesting loss or conversion of the high avidity CTL to lower avidity. Similarly, high avidity T cells maintained *ex vivo* were exquisitely sensitive to signaling with low doses of peptide (1 ng/ml) giving optimal TCR stimulation and resulting in retained avidity, proliferation and ability to kill specific targets. In contrast, high avidity T cells maintained *ex vivo* with supraoptimal TCR stimulation (10 µg/ml peptide) resulted in reduced avidity and failure to kill tumor cells. They also failed to proliferate, showed a significant increase in apoptosis and expressed high levels of the exhaustion marker programmed death-1 (PD-1) and low levels of the lymphocyte-activation gene 3 (LAG-3). This suggests high avidity T cells are recruited to the memory pool but can be lost by supraoptimal stimulation *in vitro* and *in vivo*. This is characterized by loss of function and an increase in cell death. The remaining CTL, exhibit low functional avidity that is reflected in reduced anti-tumor activity. This could contribute to failure of the immune system to control the growth of tumors and has implications for vaccination strategies and adoptive transfer of T cells.

## Introduction

It is widely accepted that the generation of high frequency T cell responses is not necessarily an indication of the induction of an effective immune response. It is apparent from previous published work that T cell functional avidity is a better indicator of clinical response [1], [2], [3], [4], [5]. The term functional avidity is often confused with affinity. Affinity is most often classified as a measure of the strength of binding of the peptide MHC molecule to the T cell receptor (TCR) whereas functional avidity is a measure of the combination of stimulation via TCR, co stimulatory molecules, adhesion molecules and cytokines and is indicative of the overall strength of interaction between T cell and target [6]. In both viral infection and tumor models, only high avidity cytotoxic T lymphocytes (CTL) mediate viral clearance and tumor eradication [1], [3], [7], [8], [9]. During the generation of an immune response *in vivo* CTL can show a range of functional avidities both at the clonal and polyclonal level. Although avidity has been shown to be important in both viral and tumor settings, the mechanisms by which high and low avidity CTL are generated *in vivo* remains unclear as the TCR cannot undergo somatic hypermutation. It has been demonstrated *in vitro* that culturing of TCR transgenic CTL in the presence of high or low dose of antigen leads to polarization of low and high avidity responses respectively [1], [3]. Evidence for polarisation of polyclonal immune responses is becoming more apparent *in vivo.* There is a growing body of information suggesting that CTL undergo clonal exhaustion *in vivo* leading to the anergy and deletion of vital antigen specific CTL [10]. This is especially common in chronic viral infections where antigen is often expressed for prolonged time periods [11], [12]. This clonal exhaustion is believed to be a result of antigen-dependent apoptosis of CTL [13]. High avidity CTL have been shown to be more sensitive to antigen dose and therefore could be subject to negative regulation by supraoptimal antigen levels and persistence of antigen *in vivo*.

We have previously shown that immunization with a DNA vaccine, that encodes tumor peptides within the complementarity determining regions (CDRs) of an antibody, results in high avidity T cells to a range of encoded peptides whereas peptide immunization results in lower avidity responses [14]. This has enabled us to use this model to further study the role of high avidity response in tumor immunity. In this study we hypothesize that high avidity anti-tumor CTL can be generated and efficiently recruited to the memory pool but that they can be subsequently impaired by supraoptimal TCR signaling.

## Materials and Methods

### Ethics Statement

Animal work was carried out under a Home Office approved project license.

### Reagents

RPMI-1640, fetal bovine serum (FBS), phosphate buffered saline (PBS), penicillin-streptomycin, HEPES, glutamine, 2-mercaptoethanol, lipopolysaccharide (LPS), Carboxyfluorescein succinimidyl ester (CFSE) and complete/incomplete Freund’s adjuvant (FA) were obtained from Sigma (Poole, UK). Murine cytokines were obtained from Peprotech EC (London, UK). Dextran sulphate was obtained from Pharmacia (Milton Keynes, UK). Fluorochrome conjugated antibodies targeting CD62L (clone MEL-14), CD127 (clone A7R34), CD86 (clone GL1), CD80 (clone 16-10A1), CD11c (clone N418), PD-1 (clone J43) and MHC class I (clone 28-14-8) were obtained from eBiosciences (San Diego, USA). Unconjugated antibody to LAG-3 (clone C9B7W) was obtained from eBioscience and used in conjunction with an anti-Rat IgG FITC conjugated secondary antibody also from eBioscience. PE-Alexa-647 or FITC labeled antibody targeting CD8 (clone KT15) was obtained from AbD Serotec (Oxford, UK) and PE labeled H-2 Kb SVYDFFVWL pentamer from ProImmune (Oxford, UK). Synthetic peptides were obtained from Peptide Synthetics (Cambridge, UK).

### Cell Lines

B16F1, MeWo and RMAS cell lines were obtained from the ATCC and maintained in RPMI with 10% FBS. Media used for splenocyte culture was RPMI-1640 with 10% FBS, 2 mM glutamine, 20 mM HEPES buffer, 100 units/ml penicillin, 100 µg/ml streptomycin (complete media) and 10^−5^M 2-mercaptoethanol.

### Mice and Immunizations

Female C57Bl/6 (Charles River, Kent, UK) mice were used between 6 and 12 weeks of age. Synthetic peptide SVYDFFVWL (Tyrosinase relate protein (TRP2) 180–188) was emulsified with complete (CFA for prime) or incomplete Freund’s adjuvant (IFA for subsequent boosts) and delivered subcutaneously (s.c.) at 25 µg/immunization. Human IgG1 antibody DNA encoding a TRP2 epitope within CDRH2 was coated onto 1.0 µm gold particles (BioRad, Hemel Hempstead, UK) using the manufacturer’s instructions and administered intradermally by the Helios Gene Gun (BioRad). Each mouse received 1 µg DNA/immunization into the shaved abdomen. Mice were immunized at days 0, 7 and 14 with DNA or peptide boosts where stated. Spleens were removed at day 20, 48 or 70 for analysis. For tumor challenge studies mice were immunized at days 0, 7, and 14 with DNA and boosted at day 64 with DNA or peptide. At day 70, 2.5×10^4^ B16F1 cells were implanted s.c. and growth monitored at 3–4 day intervals using a caliper.

### Elispot and Elisa Assays

*Ex vivo* elispot assays were performed using murine IFNγ capture and detection reagents according to the manufacturer’s instructions (Mabtech AB, Nacka Strand, Sweden). In brief, anti-IFNγ antibodies were coated onto wells of 96-well Immobilin-P plate and triplicate wells were seeded with 5×10^5^ splenocytes in complete media plus 2-mercaptoethanol. Synthetic peptide SVYDFFVWL (TRP2) at a variety of concentrations or tumor cells at 5×10^4^/well in complete media plus 2-mercaptoethanol were added to these wells and incubated for 40 hrs at 37°C. Following incubation, captured IFNγ was detected by a biotinylated anti-IFNγ antibody and development with a streptavidin alkaline phosphatase and chromogenic substrate. Spots were analyzed and counted using an automated plate reader (CTL Europe GmbH, Aalen, Germany). For elispot assays on CTL lines, triplicate wells were seeded with 5×10^4^ CTL lines in complete media. Synthetic TRP2 peptide (at a variety of concentrations) was pulsed onto RMA-S cells for 1½ hrs at 37°C and added to wells at 5×10^3^/well in complete media. Tumor cells at 5×10^3^/well were added to appropriate wells and plates incubated for 20 hrs at 37°C prior to development as detailed above. Anti-CD3 stimulation of CTL lines (5×10^4^/well) was performed in 96 well plates previously coated with anti-CD3 antibody at different concentrations and supernatant analyzed for presence of IFNγ by elisa assay after 20 hrs at 37°C. Functional avidity was calculated as the concentration mediating 50% maximal effector function using a graph of effector function versus peptide concentration.

### CTL Stimulation *in vitro* and Proliferation

Six days following the final immunization, splenocytes (5×10^6^/ml) were isolated and co-cultured at 37°C with syngeneic, irradiated (3000rads), peptide-pulsed LPS blasts in complete media plus 2-mercaptomethanol. Before use stimulator cells were labeled with relevant concentration of synthetic peptide at concentration of 2×10^7^/ml for 1 hr at 37°C in RPMI-1640. For proliferation assays red blood cells (RBCs) were lysed and splenocytes were labeled with 0.5 µM CFSE prior to culture. LPS blasts were obtained by activating splenocytes (1.5×10^6^ cells/ml) with 25 µg/ml LPS and 7 µg/ml dextran sulphate in complete media for 48 hrs at 37°C. Cultures were assayed for cytotoxic activity, IFNγ release or proliferation on day 6 in a ^51^Cr-release assay, elispot/elisa assay or by flow cytometry.

### ^51^Cr-release Assay

Target cells were labeled for 90 mins with 1.85 MBq sodium (^51^Cr) chromate (Amersham, Essex, UK) and plated at5×10^3^ targets/well in 96-well V-bottomed plates. These were co-incubated with different densities of effector cells in a final volume of 200 µl complete media. After 4 hrs at 37°C, 50 µl of supernatants were removed from each well and transferred to a Lumaplate (Perkin Elmer, Wiesbaden,Germany). Plates were read on a Topcount Microplate Scintillation Counter (Packard). Percentage specific lysis was calculated using the following formula: specific lysis  = 100×[(experimental release-spontaneous release)/(maximum release-spontaneous release)].

### Flow Cytometry

Staining of splenocytes *ex vivo* was performed by lysing RBCs using RBC lysing solution (Sigma) according to manufacturers guide lines. No RBC lysis was performed before staining of *in vitro* cultured CTL. Cells were subsequently stained with Phycoerythrin (PE) labeled H-2 Kb SVYDFFVWL (TRP2 180–188) pentamer at room temperature in the dark for 15 mins followed by incubation with other cell surface markers CD8-PE-Alexa-647 or FITC, CD62L-FITC, CD127-PECy7, PD-1 PE-Cy7 or LAG-3 for 30 mins on ice. Viability stain 7-AAD (eBioscience) was added at room temperature in the dark for 10mins immediately prior to analysis. For staining of LPS blasts, cells were stained with CD11c-PECy5, MHC I FITC, CD80 PE or CD86 PE for 30mins on ice. Data acquisition and analysis was performed on a FC500 flow cytometer (Beckman Coulter).

### Statistics

Comparative analysis of the Elispot, flow cytometry, tumor volumes and CTL lysis data was performed by applying the student’s t-test with values of p calculated accordingly. Comparison of avidity curves was performed by applying the F test and survival analysis by applying the Log-rank test using Graphpad Prism software.

## Results

We have previously demonstrated that immunization with epitopes engineered within a human antibody framework induces high avidity CTL [14]. We have used this model to investigate if high avidity T cells are recruited to the memory pool and if they are acutely sensitive to TCR signaling.

### DNA Immunization Induces CTL with High Functional Avidity whereas Peptide Immunization Elicits Low Avidity, Functionally Impaired CTL

**Figure 1:**
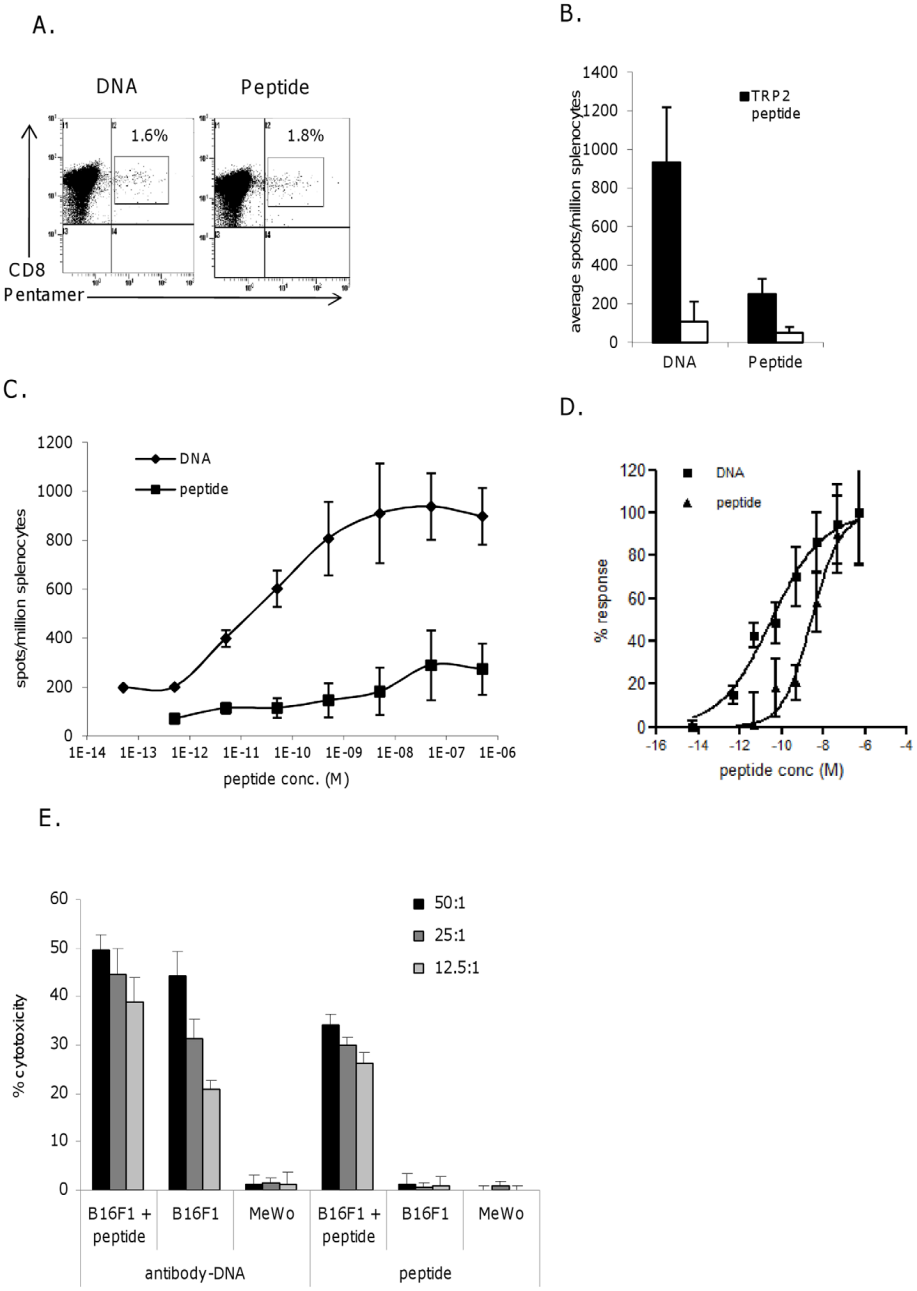
Peptide immunization induces low avidity functionally impaired CTL. Splenocytes from mice immunized with peptide or DNA were analyzed at day 20 for A, Presence of antigen specific CTL by pentamer and CD8 staining. B, Frequency of epitope specific responses in IFNγ elispot assay C, Avidity and frequency of epitope specific responses by measuring responses to increasing peptide concentration in IFNγ elispot assay, D, Analysis of the avidity by normalization of responses. E, The ability of CTL lines to lyse tumor cells in chromium release assay. Data is representative of at least three independent experiments.

C57Bl/6 mice were immunized with either a DNA construct containing the H-2 Kb restricted SVYDFFVWL epitope from TRP2 antigen within the CDRH2 region of an antibody or the same epitope as a peptide. Peptide immunized mice showed similar frequencies of antigen specific CD8 cells to DNA immunized mice by pentamer staining (Figure 1a) and by IFNγ elispot assay (Figure 1b). In contrast, mice immunized with antibody-DNA induced over a 100 fold higher avidity response than mice immunized with peptide (p = 0.0008) (Figure 1c,d). This difference in functional avidity was reflected in tumor cell killing. CTL were tested for lysis of B16F1 tumor cells, those derived from antibody-DNA immunized mice were capable of efficient killing whereas those derived from peptide immunized mice were not (Figure 1e).

### High Avidity Responses are Efficiently Maintained into Memory

**Figure 2:**
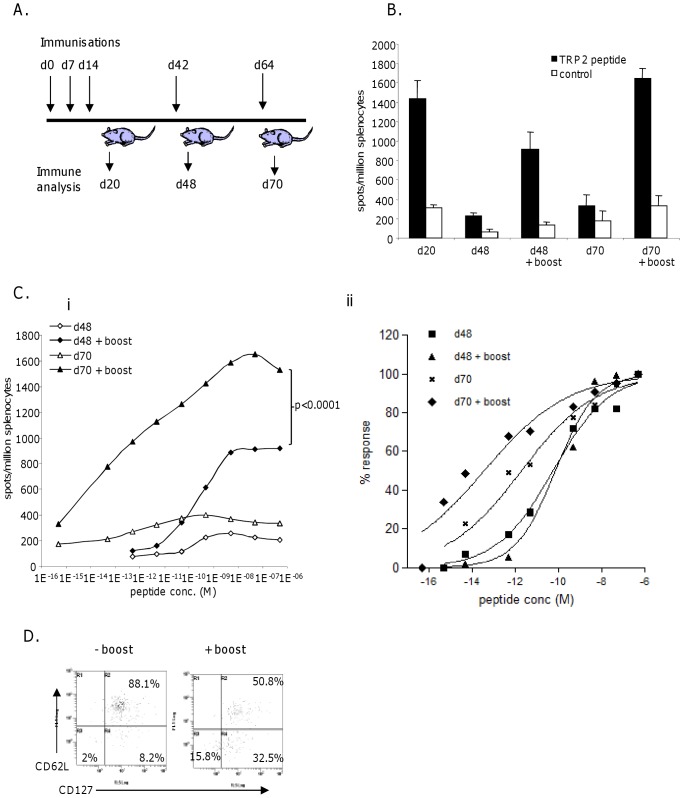
High avidity responses induced by DNA immunization are efficiently maintained into memory. A, schematic of immunization regime. B, Mice immunized with DNA were boosted at days 42 or 64 and frequency of immune responses analysed in IFNγ elispot assay at days 20, 48 and 70. C, Analysis of avidity of responses by peptide titration in IFNγ elispot assay, D, normalization of responses to increasing peptide concentration. E, Analysis of memory phenotype of antigen specific CTL by combination staining for CD62L and CD127 markers. Data is representative of at least three independent experiments.

To determine if high avidity responses induced by antibody-DNA immunization were maintained into memory, splenocytes from immunized mice were analyzed for the frequency and avidity of functional epitope specific immune responses by IFNγ elispot assay, 48 and 70 days post immunization. Parallel groups of mice were boosted at 42 or 64 days post immunization with a single dose of antibody-DNA to determine the effect of booster immunization (Figure 2a). At 48 days following immunization low level TRP2 epitope specific responses (three fold over background) were observed which significantly expanded six fold over background upon booster immunization (Figure 2b). Responses detectable at 48 days were of high functional avidity (10^−10^ M peptide) (Figure 2c). Analysis of immune responses at 70 days revealed a similar frequency as at 48 days. However, these responses showed higher functional avidity (<10^−12^ M) than those analyzed at earlier time points (p<0.0001), suggesting that the progression into memory selects for and retains the higher avidity T cells. This response was boosted into even higher avidity (<10^−13^M) by DNA immunisation.

To assess the memory phenotype of these high avidity responses, splenocytes from mice taken at 70 days post immunization, were also analyzed for the expression of the memory markers, CD62L and CD127 (IL-7Rα) on TRP2 pentamer stained CD8 T cells. Combined analysis of CD62L and CD127 expression on TRP2 specific CD8 cells shows cells are mainly of the central memory phenotype (CD62L+ CD127+) with a smaller proportion of effector memory (CD62L- CD127+) and effector cells (CD62L- CD127-) (Figure 2e). Following booster immunization at day 64 a higher frequency of effector and effector memory phenotype are observed although a significant central memory population is retained.

### High Avidity Memory Responses are Lost by Booster with Peptide Immunogen *in vivo*

**Figure 3:**
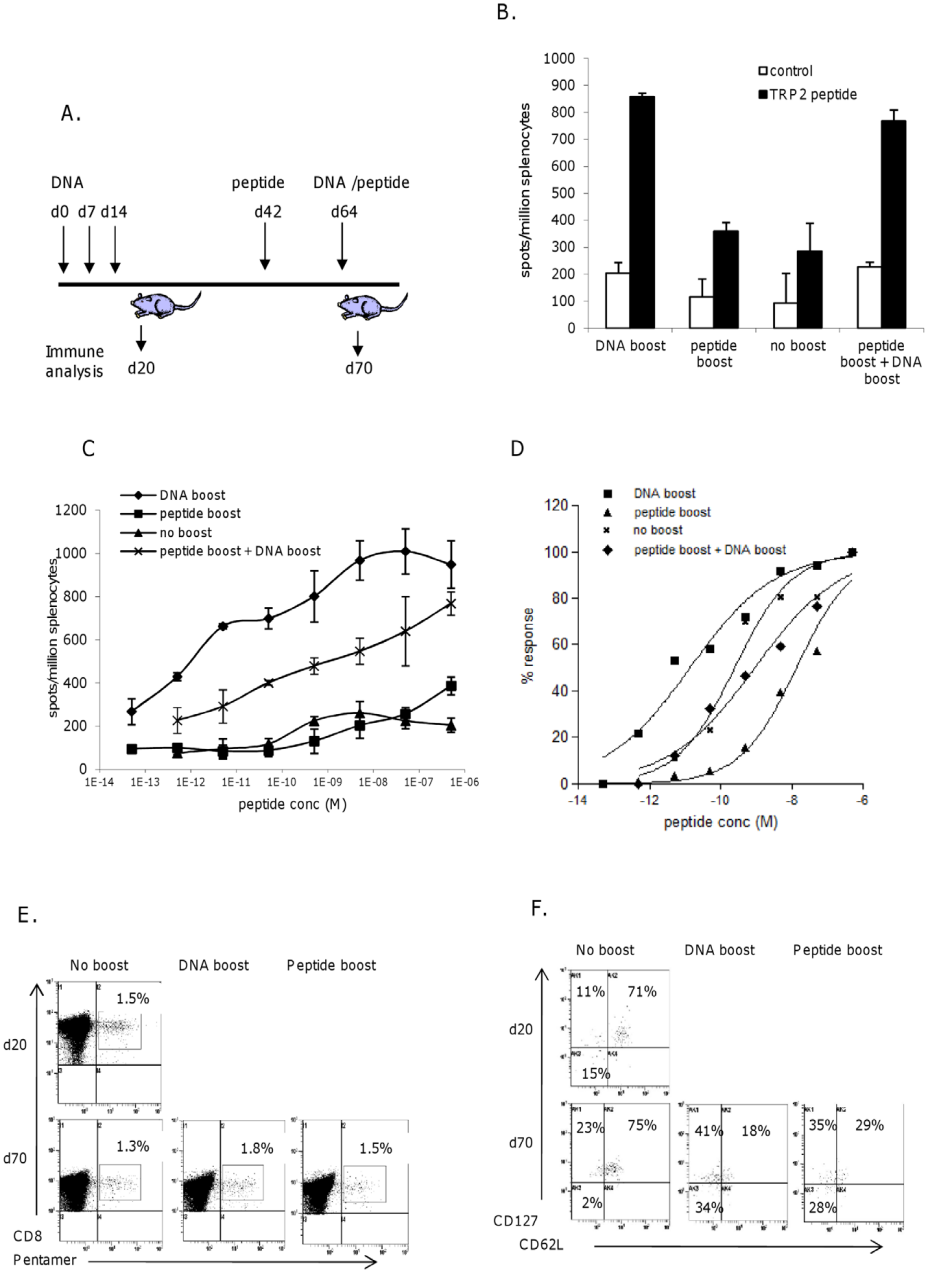
Peptide boost leads to loss of high avidity responses *in vivo*. A, schematic of immunization regime. Mice immunized with DNA were boosted at day 63 with DNA or peptide, or with peptide at day 42 followed by DNA at day 63. Responses were analyzed at day 70 for frequency (B) and avidity (C) by measuring responses to increasing peptide concentration in IFNγ elispot assay, D) normalization of responses to increasing peptide concentration in IFNγ elispot assay. E, Analysis of antigen specific CTL by pentamer and CD8 staining of immunized splenocytes. F, Analysis of memory phenotype of antigen specific CTL by combination staining for CD62L and CD127 markers. Data is representative of at least three independent experiments.

Since peptide immunogen has been shown to induce low avidity responses *in vivo*. It was examined if high avidity responses induced by antibody-DNA could be influenced by subsequent exposure to peptide immunogen. Mice were immunized with antibody-DNA and responses left to establish into memory. At day 64 without any boost they had a functional avidity of 1E^−10^M they were then boosted *in vivo* with peptide (Figure 3a). Boosting of high avidity memory CTL responses using peptide immunogen revealed an increase in the frequency of the response compared to no boost (Figure 3b) with a 100 fold reduction in functional avidity (2E^−08^M, p<0.0001) (Figure 3c,d) compared to no boost and a 5,000 fold reduction in avidity when compared to a DNA boost (2E^−11^M, p<0.0001) (Figure 3c,d). Pentamer staining of splenocytes from these mice reveals similar numbers of antigen specific CD8s in each group (Figure 3e). Antigen specific CD8s also show similar frequency of effector and memory phenotypes upon booster immunization (Figure 3f).

To determine if this lower avidity response could be reactivated to high avidity, mice were further boosted with antibody-DNA (Figure 3c,d). Boost, with antibody-DNA following the peptide challenge showed restimulation of high frequency epitope specific CTL (Figure 3b). However, this response remained of similar functional avidity when compared to mice that had not received a peptide boost suggesting that the DNA could still selectively stimulate any remaining higher avidity T cells.

### Low Avidity Responses Resulting from Peptide Boost Show Limited Anti-tumor Efficacy *in vitro* and *in vivo*

**Figure 4:**
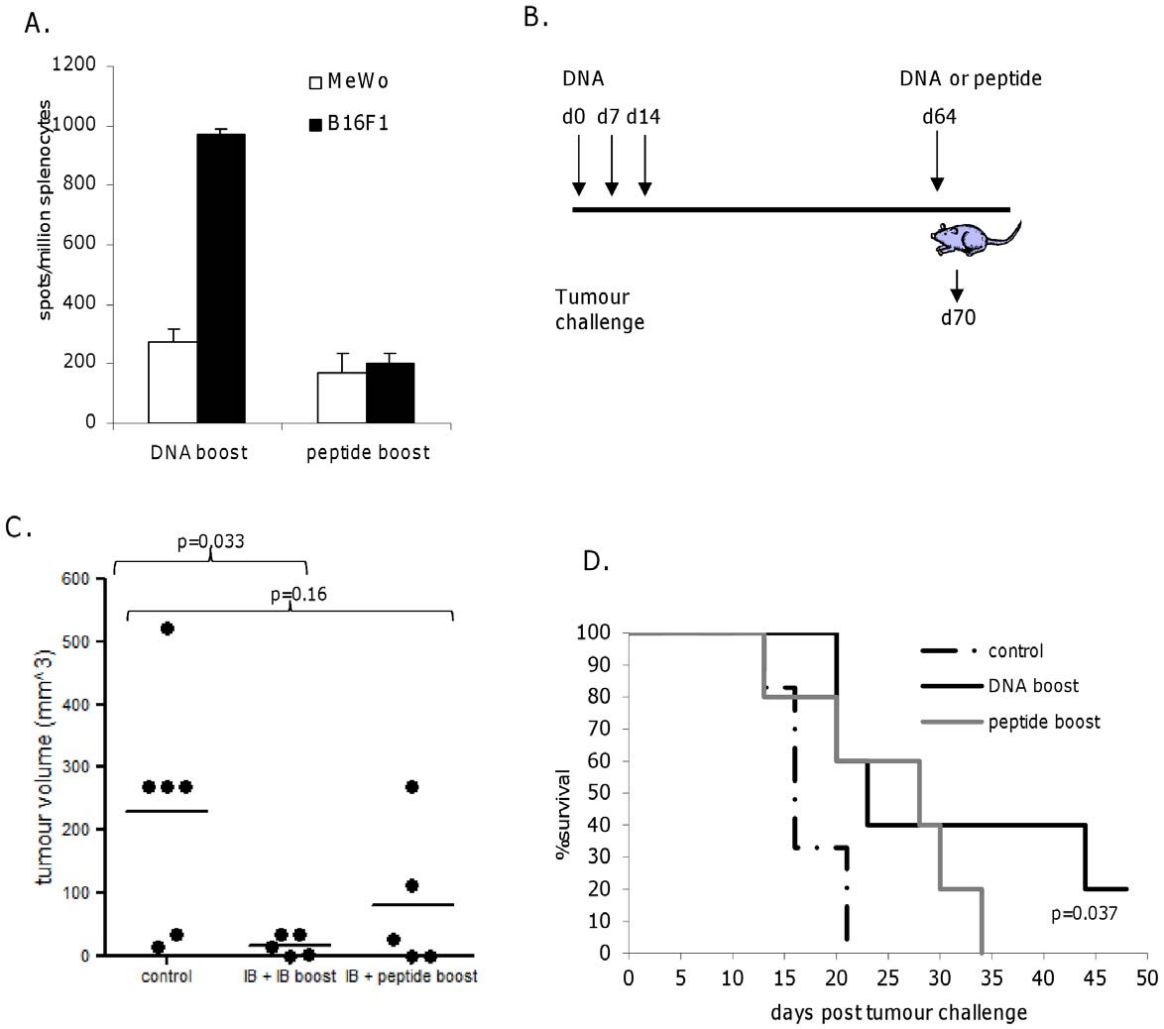
Low avidity responses show limited anti-tumor efficacy. A, Recognition of B16F1 and MeWo cells by immunized splenocytes in IFNγ elispot assay. B, schematic of immunization regime. C, Tumor volume at day 16 post tumor challenge. D, Survival analysis of mice immunized with DNA and boosted with DNA or peptide followed by challenge with B16F1 tumor.

This low avidity response induced as a result of boosting high avidity memory responses with peptide was analyzed for its ability to recognize the syngeneic B16F1 melanoma cell line expressing TRP2 *in vitro* compared to the HLA mismatched MeWo control. Responses from mice boosted with antibody-DNA that retain high avidity show recognition of B16F1 cells whereas low avidity responses from those boosted with peptide are incapable of specific tumor cell recognition (Figure 4a). To analyse the effect of low avidity responses *in vivo* mice boosted with antibody-DNA or peptide were challenged with B16F1 tumor (Figure 4b). Mice with high avidity responses (10^−12^ M) that were boosted with antibody-DNA show significantly delayed tumor growth (p = 0.03) at day 16 post tumor implant compared to control. This is reflected in significantly enhanced survival (p = 0.037) in this group compared to control (Figure 4d).

Although the peptide boosted mice did show some anti-tumour effects this did not reach significance due to the lower avidity (Figure 4c).

### Supraoptimal TCR Stimulus Results in Loss of High Avidity Response *in vitro*

**Figure 5:**
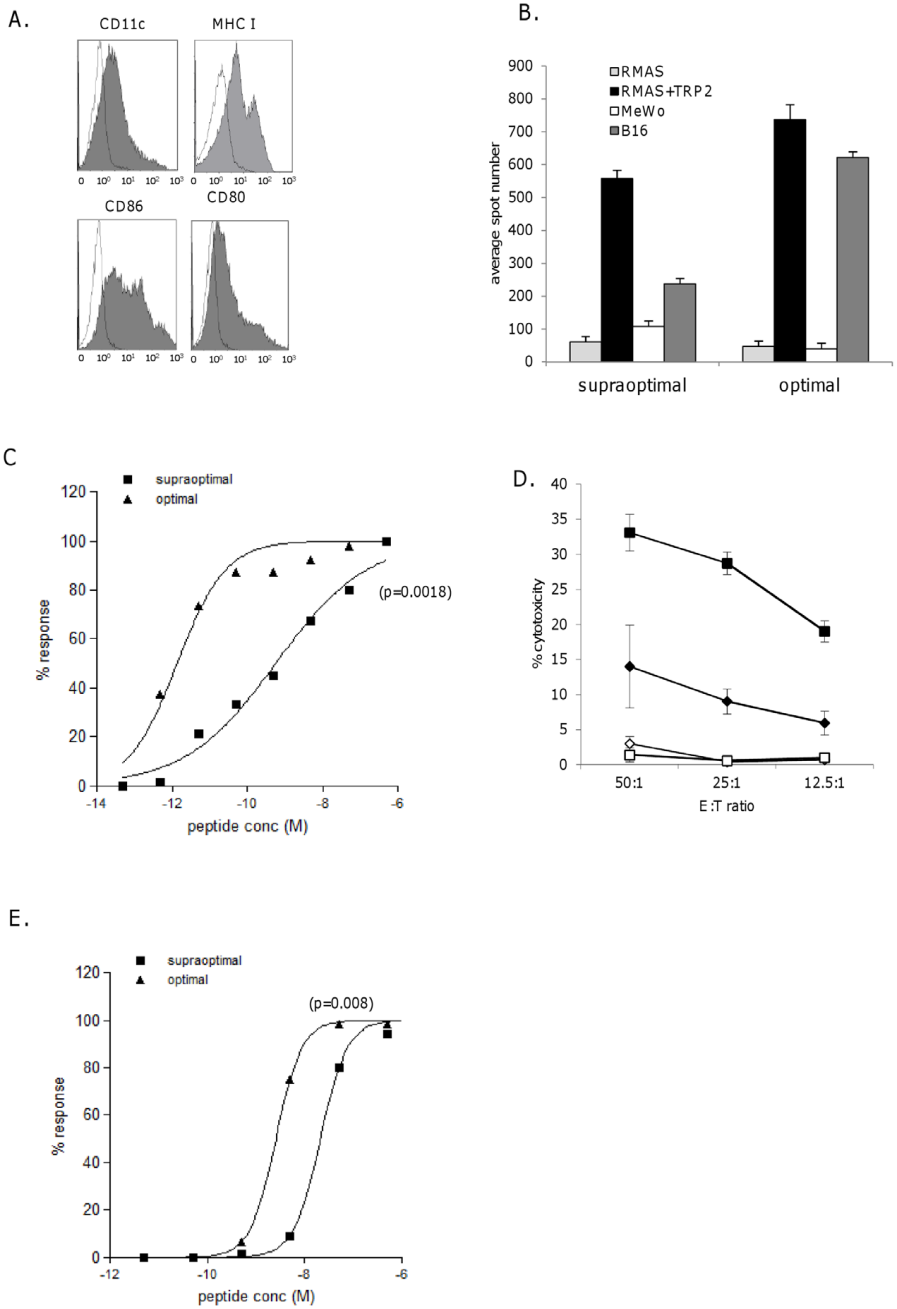
High avidity CTL responses can be modulated by the doses of TCR stimulus. A, LPS blasts were stained for expression of CD11c, MHC class I, CD80, CD86 (filled histograms) compared to control (open histograms). B, High avidity CTL from DNA immunization were stimulated *in vitro* with supraoptimal (100 µg/ml) and optimal (10 ng/ml) dose peptide pulsed LPS blasts. After 6 days *in vitro* cultures were assessed for peptide specific responses against peptide (1 µg/ml) pulsed RMAS cells and tumor cell recognition. C, i) CTL cultures were analyzed for avidity of epitope specific responses by measuring responses to increasing peptide concentration on RMAS cells in IFNγ elispot assay, ii) normalization of responses. D, supraoptimal (diamonds) and optimal (squares) dose peptide stimulated CTL were assayed for cytotoxicity against B16F1 (closed symbols) or MeWo (open symbols) by chromium release assay at 50∶1, 25∶1 and 12.5∶1 effector:target ratios. Data is representative of at least three independent experiments. E, CTL cultures stimulated with supraoptimal and optimal dose peptide pulsed LPS blasts were analyzed for sensitivity to CD3 stimulation in IFNγ elisa assay (normalized data is shown). Data is representative of at least three independent experiments.

To assess if high avidity responses were differentially affected by TCR signaling, high avidity CTL responses were subjected to a short *in vitro* culture with supraoptimal (10 µg/ml) and optimal (1 ng/ml; Ic50 of high avidity T cells) dose of peptide pulsed on LPS blasts. These stimulated antigen presenting cells (APCs) express high levels of costimulatory molecules and therefore provide optimal costimulation for CTL responses (Figure 5a). After 6 days stimulation, resulting CTL lines were tested for functional epitope specific response and tumor cell recognition in elispot assay. Figure 5b shows stimulation of the high avidity response *in vitro* with high or low dose peptide maintained a similar frequency of peptide specific responses. In contrast, analysis of functional avidity revealed that optimal stimulation (low dose) retained avidity whereas supraoptimal stimulation (high dose) resulted in significantly lower avidity (p = 0.0018) (Figure 5c). The higher functional avidity of the optimally stimulated CTL was reflected in the ability of these cells to recognize and kill the B16F1 tumor cells (p = 0.022) (Figure 5b and d). The activity of the high avidity T cells suggests higher sensitivity to TCR triggering than seen in low avidity T cells. This was supported by measuring responses of these cells to CD3 stimulation. High avidity CTL were more sensitive to lower doses of anti-CD3 compared to low avidity T cells (p = 0.008) (Figure 5e).

### High dose TCR Stimulus Promotes T Cell Impairment and Death *in vitro*

**Figure 6:**
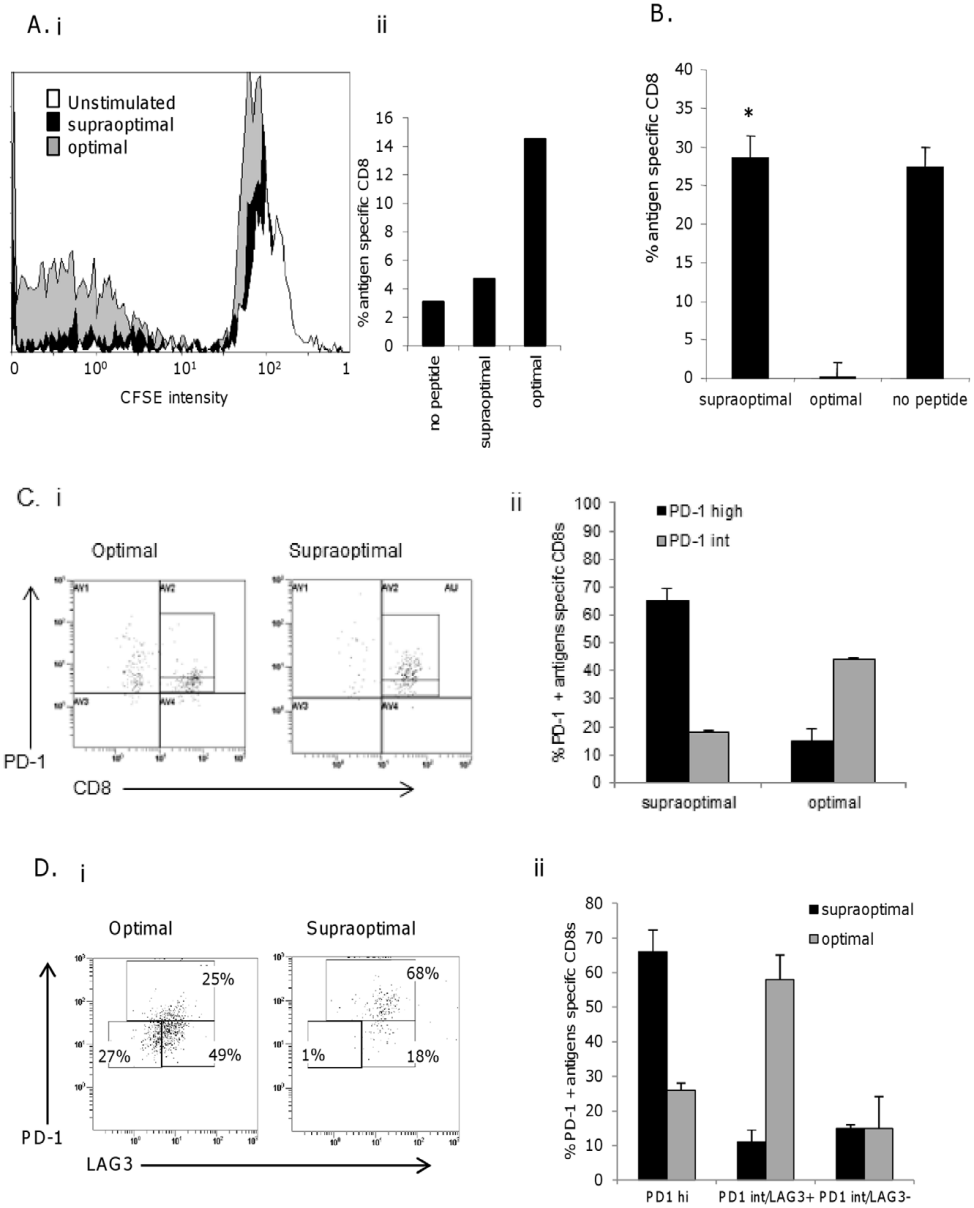
Supraoptimal dose antigen promotes T cell impairment and death. Splenocytes from DNA immunization were stimulated *ex vivo* with supraoptimal (100 µg/ml) and optimal (10 ng/ml) dose peptide pulsed LPS blasts. A, CFSE labeled splenocytes were assayed for proliferation of antigen specific CTL after 6 days *in vitro* culture. CFSE intensity is shown gated on pentamer positive CD8 cells. CTL were stained for B, the uptake of 7-AAD (plots gated on Pentamer+ CD8+ cells) * p = 0.0093, C, the expression of PD-1 (plots gated on Pentamer+ cells), D) average data and E, expression of PD-1 and LAG-3 (plots gated on Pentamer+ CD8+ cells), F) average data. Data is representative of at least three independent experiments.

The loss of functional avidity with supraoptimal stimulation may suggest that overstimulation of TCR can lead to cellular exhaustion, cell death or a combination of both. Analysis of proliferation of high avidity CTL stimulated with high and low dose peptide demonstrated that the low, optimal dose of peptide induced better proliferation than the higher dose of peptide (Figure 6a) resulting in a higher percentage of pentamer positive cells (14.5%) compared to cells stimulated with supraoptiomal doses (5%; Figure 6a ii). Supraoptimal, high dose peptide shows CFSE dilution in only 5.2% of pentamer positive CD8s as opposed to 62.5% dilution with optimaldose peptide. Unstimulated cells show 2.6% of pentamer positive CD8s with dilution in CFSE staining intensity. High avidity CTL lines exposed to optimal and supraoptimal doses of peptide were also were examined for the extent of cell death by 7-AAD uptake. Figure 6b shows that stimulation of the CTL with high supraoptimal dose of peptide leads to more 7-AAD positive antigen specific CTL than lines stimulated with low optimal dose peptide (p = 0.0093). This suggested a higher rate of cell death amongst those stimulated with high supraoptimal dose peptide. To examine the possibility of exhaustion cell lines were analyzed for the markers PD-1 and LAG-3 after optimal and supraoptimal peptide stimulation. Expression of PD-1 is known to be upregulated upon activation and it has been suggested that predominantly CD8 cells expressing high levels of PD-1 are those that are functionally inert. Cells lines stimulated supraoptimally showed larger numbers of antigen specific CD8s expressing high levels of PD-1 than those with optimal stimulation (Figure 6c,d). More recently cells expressing intermediate levels of PD-1 have been shown to retain functional capability with those that also express the marker LAG-3 being most responsive [15]. Staining of optimal and supraoptimal stimulated cultures for PD-1 and LAG-3 reveals more of PD-1 intermediate cells in optimally stimulated cultures to express LAG-3 (Figure 6e,f).

## Discussion

High avidity T cells have been shown to be vital for both anti-tumor immunity and viral clearance. Using the TRP2, CD8 epitope SVYDFFVWL, as a model self-epitope in mice and the ability to induce high avidity CTL by encoding this epitope within an antibody-DNA construct, we have shown that high avidity CTL are predominantly recruited to the memory response. Analysis of immune responses at 70 days revealed a similar frequency as at 48 days. However, the day 70 responses showed remarkable picomolar avidity to a self-antigen which was 100 fold higher than those seen at earlier time points. Others have suggested that the progression into memory selects for and retains the higher avidity T cells [16], [17]. In our study we generated CTL responses with picomolar avidities which caused vitiligo and tumor rejection. One possible mechanism for progressive selection of high avidity T cells is that high avidity T cells are more sensitive to IL-15 driven homeostatic proliferation in the absence of antigen [18]. This may explain the increase in avidity seen in our study following a boost at d70, as the antigen from our DNA vaccine persists at the site of injection for 35 days but is undetectable even by PCR by day 90 (unpublished results). These highly functional CTL were able to reject a tumor challenge in 20% of animals. There is some controversy in the literature as to whether high avidity T cells are preferentially selected into memory. Higher avidity responses in memory than during the primary response have been shown [19]. However, these were in response to a viral prime and challenge and it was unclear if the avidity was based upon epitope selection. In our study, we focused on the avidity of the response to a single, self epitope and showed a remarkable increase in avidity. Turner *et al*. showed that whereas the avidity of the memory response to OVA encoded within viruses increased in wild type mice, avidity maturation was self limited in mice that express OVA as a self antigen [20]. They also failed to see autoimmunity.

The high avidity CTL memory responses could be efficiently boosted by DNA immunization. In contrast, the functional capability of the high avidity memory response was dramatically reduced upon boosting *in vivo* with peptide immunogen. If this was a result of the peptide attenuating the high avidity memory response, then a subsequent boost with DNA should not recover the avidity. This was indeed the case as antibody-DNA, restimulated the low avidity T cells to a higher frequency but could only partially recover the avidity of the original DNA prime and was very poor in comparison to a DNA boost in the absence of peptide immunisation. This suggests that the peptide boost had deleted/impaired the original high avidity memory response. It has been suggested that CD8 tolerance/anergy can be induced by peptide immunization specifically when multiple doses are given [21], [22], [23], [24]. It is thought that this is due to the presentation of these peptides on non professional APCs or long term systemic presentation of the epitope [25]. A report from Rezvani *et al.* highlights this in a clinical setting where patients receiving repeated immunizations with peptide in montanide adjuvant, showed loss of high avidity CTL responses correlating with lack of anti-tumor efficacy [26]. However the fact that the DNA boost appears to correct, at least in part, the low avidity effect of the peptide boost is an exciting result, suggesting that even in the face of a very low avidity population, the DNA vaccination can still move the response towards higher avidity.

It has previously been suggested that high avidity T cells are highly susceptible to signaling via MHC:peptide due to the assimilation of the TCR into preformed signaling rafts which can rapidly amplify signal [27], [28]. The functional avidity of CTL has been shown to be linked to the surface expression of CD8, engagement of CD3 and signal transduction following TCR engagement with peptide MHC. High avidity CD8 cells are known to express higher levels of CD8 and show clustering of signaling molecules into lipid rafts resulting in lower activation thresholds and stronger stimulation signals from TCR:peptide MHC complexes [28], [29], [30]. To determine if the high avidity T cells were attenuated by supraoptimal signaling, the high avidity T cells were stimulated *in vitro* with different doses of TCR signaling. The use of LPS blasts provided high levels of costimulation so as to solely examine the effect of TCR signal strength on restimulation of responses.

Stimulation of a high avidity response *ex vivo* with optimal doses of peptide, induces proliferation and maintains their potent avidity and killing function. Thus high avidity memory T cells will be acutely sensitive to further encounter with low dose antigen, either low viral infection or early tumor development. In contrast, stimulation of a high avidity response *ex vivo* with high supraoptimal dose of peptide immunogen resulted in low avidity responses and loss of functional capability *in vitro*. This suggests that the strength of the TCR signal received plays a major role in restimulation of responses. High dose peptide stimulus *in vitro* appeared to induce less proliferation and increased cell death which is consistent with reports of T cells pushed to exhaustion. This is consistent with the hypothesis of others that over stimulation through TCR:peptide MHC complex pushes high avidity CTL towards apoptosis [13], [31], [32]. Interestingly, a recent study by Muraoka *et al.* demonstrated the apoptosis of epitope specific CTL upon repeat peptide vaccination [24].

The inhibitory receptor PD-1 is known to be upregulated upon T cell activation and the extent of engagement of PD-1 by its ligands is known to regulate the threshold for T cell activation [33], [34], [35], [36]. It is also a marker that has been associated with functional exhaustion of CD8 T cells. CTL cultures in this study stimulated with optimal and supraoptimal peptide dose both show expression of PD-1. However, the level of PD-1 expression differs dramatically with supraoptimal stimulated CTL expressing higher levels of the marker. Increased expression of PD-1 has been demonstrated on antigen specific CD8 cells induced by peptide immunization which exhibited low *in vivo* cytotoxicity and on exhausted CTL [37], [38]. More recently it has also been documented that expression of high levels of PD-1 by tumor infiltrating lymphocytes correlates with functional impairment and suggests a role for PD-1 ligands in combination with prolonged antigen expression by tumors in establishment of T cell anergy [39], [40], [41]. Supraoptimal TCR stimulus and high level of PD-1 expression are therefore likely to lead to cell death and exhaustion and would help explain the loss of high avidity responses. Future studies to assess if blockade of the PD-1 pathway restores proliferation and prevents cell death will be undertaken. It has been suggested that expression of PD-1 alone cannot be taken as a marker of functionally exhausted cells. Other markers such as LAG-3 are also upregulated upon T cell activation and associated with negative regulation [42]. A study by Grosso *et al*. on chronically stimulated CD8 T cells interestingly discovered that the presence of LAG-3 does not always correlate with a decrease in function. Those cells expressing low levels of PD-1 in combination with LAG-3 correlated with increased functional ability which is a phenotype observed in the majority of optimally stimulated CTL cultures in this study [15]. However, the functional ability of cells expressing high levels of PD-1 was impaired independent of the LAG-3 status.

This study highlights the importance of optimal stimulation for the *in vivo* induction and maintenance of high avidity CTL responses. In contrast, supraoptimal stimulation can lead to non productive immune responses. This has implications for tumor therapy as high dose sustained TCR stimulation either by inappropriate vaccination or by tumor cells presenting cognate peptide:MHC in the absence of costimulation could lead to selection of low avidity T cells that fail to control tumor growth.

## References

[1] Alexander-Miller MA, Leggatt GR, Berzofsky JA (1996). Selective expansion of high- or low-avidity cytotoxic T lymphocytes and efficacy for adoptive immunotherapy.. Proc Natl Acad Sci U S A.

[2] Dutoit V, Rubio-Godoy V, Dietrich P, Quiqueres A, Schnuriger V (2001). Heterogeneous T-cell response to MAG-A10_254–262_: High avidity-specific cytolytic T lymphocytes show superior antitumour activity.. Cancer Res.

[3] Zeh HJ, Perry-Lalley D, Dudley ME, Rosenberg SA, Yang JC (1999). High avidity CTLs for two self-antigens demonstrate superior in vitro and in vivo antitumor efficacy.. J Immuno.

[4] Ayyoub M, Rimoldi D, Guillaume P, Romero P, Cerottini JC (2003). Tumor-reactive, SSX-2-specific CD8+ T cells are selectively expanded during immune responses to antigen-expressing tumors in melanoma patients.. Cancer Res.

[5] Durrant LG, Pudney V, Spendlove I, Metheringham RL (2010). Vaccines as early therapeutic interventions for cancer therapy: neutralising the immunosuppressive tumour environment and increasing T cell avidity may lead to improved responses.. Expert Opin Biol Ther.

[6] Sandberg JK, Franksson L, Sundback J, Michaelsson J, Petersson M (2000). T cell tolerance based on avidity thresholds rather than complete deletion allows maintenance of maximal repertoire diversity.. J Immunol.

[7] Gallimore A, Glitheo A, Godkin A, Tissot AC, Pluckthun A (1998). Induction and Exhaustion of Lymphocytic Choriomeningitis Virus-specific Cytotoxic T Lymphocytes Visualized Using Soluble Tetrameric \Major Histocompatibility Complex Class I-Peptide Complexes.. J Exp Med.

[8] Yee C, Savage PA, Lee PP, Davis MM, Greenberg PD (1999). Isolation of high avidity melanoma-reactive CTL from heterogenous populations using peptide-MHC tetramers.. J Immunol.

[9] Sedlik C, Dadaglio G, Saron MF, Deriaud E, Rojas M (2000). In vivo induction of a high-avidity, high-frequency cytotoxic T-lymphocyte response is associated with antiviral protective immunity.. J Virol.

[10] Terheyden P, Schrama D, Pedersen LO, Andersen MH, Kampgen E (2003). Longitudinal analysis of MART-1/HLA-A2-reactive T cells over the course of melanoma progression.. Scand J Immunol.

[11] Wherry EJ, Ha SJ, Kaech SM, Haining WN, Sarkar S (2007). Molecular signature of CD8+ T cell exhaustion during chronic viral infection.. Immunity.

[12] Wherry EJ, Blattman JN, Murali-Krishna K, van der Most R, Ahmed R (2003). Viral persistence alters CD8 T-cell immunodominance and tissue distribution and results in distinct stages of functional impairment.. J Virol.

[13] Derby MA, Snyder JT, Tse R, Alexander-Miller MA, Berzofsky JA (2001). An abrupt and concordant initiation of apoptosis: antigen-dependent death of CD8+ CTL.. Eur J Immunol.

[14] Pudney VA, Metheringham RL, Gunn B, Spendlove I, Ramage JM (2010). DNA vaccination with T-cell epitopes encoded within Ab molecules induces high-avidity anti-tumor CD8+ T cells.. Eur J Immunol.

[15] Grosso JF, Goldberg MV, Getnet D, Bruno TC, Yen HR (2009). Functionally distinct LAG-3 and PD-1 subsets on activated and chronically stimulated CD8 T cells.. J Immunol.

[16] Slifka MK, Whitton JL (2001). Functional avidity maturation of CD8(+) T cells without selection of higher affinity TCR.. Nat Immunol.

[17] Rocha B, Tanchot C (2006). The Tower of Babel of CD8+ T-cell memory: known facts, deserted roads, muddy waters, and possible dead ends.. Immunol Rev.

[18] Stoklasek TA, Colpitts SL, Smilowitz HM, Lefrancois L (2010). MHC class I and TCR avidity control the CD8 T cell response to IL-15/IL-15Ralpha complex.. J Immunol.

[19] Hill AB, Blanden RV, Parrish CR, Mullbacher A (1992). Restimulated memory Tc cells have a higher apparent avidity of interaction with targets than primary virus-immune Tc cells as indicated by anti-CD8 blocking.. Immunol Cell Biol 70 (Pt.

[20] Turner MJ, Jellison ER, Lingenheld EG, Puddington L, Lefrancois L (2008). Avidity maturation of memory CD8 T cells is limited by self-antigen expression.. J Exp Med.

[21] Aichele P, Brduscha-Riem K, Oehen S, Odermatt B, Zinkernagel RM (1997). Peptide antigen treatment of naive and virus-immune mice: antigen-specific tolerance versus immunopathology.. Immunity.

[22] Aichele P, Brduscha-Riem K, Zinkernagel RM, Hengartner H, Pircher H (1995). T cell priming versus T cell tolerance induced by synthetic peptides.. J Exp Med.

[23] Toes REM, Offringa R, Blom RJJ, Melief CJM, Kast WM (1996). Peptide vaccination can lead to enhanced tumor growth through specific T-cell tolerance induction.. Proceedings of the National Academy of Science in the USA.

[24] Muraoka D, Kato T, Wang L, Maeda Y, Noguchi T (2010). Peptide vaccine induces enhanced tumor growth associated with apoptosis induction in CD8+ T cells.. J Immunol.

[25] den Boer AT, Diehl L, van Mierlo GJ, van der Voort EI, Fransen MF (2001). Longevity of antigen presentation and activation status of APC are decisive factors in the balance between CTL immunity versus tolerance.. J Immunol.

[26] Rezvani K, Yong AS, Mielke S, Savani BN, Musse L (2008). Leukemia-associated antigen-specific T-cell responses following combined PR1 and WT1 peptide vaccination in patients with myeloid malignancies.. Blood.

[27] Xavier R, Brennan T, Li Q, McCormack C, Seed B (1998). Membrane compartmentation is required for efficient T cell activation.. Immunity.

[28] Cawthon AG, Alexander-Miller MA (2002). Optimal colocalization of TCR and CD8 as a novel mechanism for the control of functional avidity.. J Immunol.

[29] Cawthon AG, Lu H, Alexander-Miller MA (2001). Peptide requirement for CTL activation reflects the sensitivity to CD3 engagement: correlation with CD8αβ versus CD8αα expression.. J Immunol.

[30] Kroger CJ, Alexander-Miller MA (2007). Dose-dependent modulation of CD8 and functional avidity as a result of peptide encounter.. Immunology.

[31] Alexander-Miller MA, Derby MA, Sarin A, Henkart PA, Berzofsky JA (1998). Supraoptimal peptide-major histocompatibility complex causes a decrease in bc1–2 levels and allows tumor necrosis factor alpha receptor II-mediated apoptosis of cytotoxic T lymphocytes.. J Exp Med.

[32] Alexander-Miller MA, Leggatt GR, Sarin A, Berzofsky JA (1996). Role of antigen, CD8, and cytotoxic T lymphocyte (CTL) avidity in high dose antigen induction of apoptosis of effector CTL.. J Exp Med.

[33] Tseng SY, Otsuji M, Gorski K, Huang X, Slansky JE (2001). B7-DC, a new dendritic cell molecule with potent costimulatory properties for T cells.. J Exp Med.

[34] Freeman GJ, Long AJ, Iwai Y, Bourque K, Chernova T (2000). Engagement of the PD-1 immunoinhibitory receptor by a novel B7 family member leads to negative regulation of lymphocyte activation.. J Exp Med.

[35] Yamazaki T, Akiba H, Iwai H, Matsuda H, Aoki M (2002). Expression of programmed death 1 ligands by murine T cells and APC.. J Immunol.

[36] Goldberg MV, Maris CH, Hipkiss EL, Flies AS, Zhen L (2007). Role of PD-1 and its ligand, B7-H1, in early fate decisions of CD8 T cells.. Blood.

[37] Liu Y, Xu L, Jiang Y, Sun J, He X (2007). Phenotypic and functional analysis of LCMV gp33–41-specific CD8 T cells elicited by multiple peptide immunization in mice revealed the up-regulation of PD-1 expression on antigen-specific CD8 T cells.. Cell Mol Immunol.

[38] Barber DL, Wherry EJ, Masopust D, Zhu B, Allison JP (2006). Restoring function in exhausted CD8 T cells during chronic viral infection.. Nature.

[39] Ahmadzadeh M, Johnson LA, Heemskerk B, Wunderlich JR, Dudley ME (2009). Tumor antigen-specific CD8 T cells infiltrating the tumor express high levels of PD-1 and are functionally impaired.. Blood.

[40] Fourcade J, Kudela P, Sun Z, Shen H, Land SR (2009). PD-1 is a regulator of NY-ESO-1-specific CD8+ T cell expansion in melanoma patients.. J Immunol.

[41] Chapon M, Randriamampita C, Maubec E, Badoual C, Fouquet S (2011). Progressive upregulation of PD-1 in primary and metastatic melanomas associated with blunted TCR signaling in infiltrating T lymphocytes.. J Invest Dermatol.

[42] Workman CJ, Cauley LS, Kim IJ, Blackman MA, Woodland DL (2004). Lymphocyte activation gene-3 (CD223) regulates the size of the expanding T cell population following antigen activation in vivo.. J Immunol.

